# Purification and Characterization of a Novel Extracellular Haloprotease Vpr from *Bacillus licheniformis* Strain KB111

**DOI:** 10.17113/ftb.60.02.22.7301

**Published:** 2022-06

**Authors:** Tita Foophow, Duangjai Sittipol, Neeranuch Rukying, Weerachon Phoohinkong, Nujarin Jongruja

**Affiliations:** 1 Department of Nutrition and Culinary Arts for Health Capability and Anti-Aging Wellness, School of Culinary Arts, Suan Dusit University, Dusit, 10300 Bangkok, Thailand; 2 Department of Microbiology, Faculty of Science, King Mongkut’s University of Technology Thonburi, Thung Khru, 10140 Bangkok, Thailand; 3 Faculty of Science and Technology, Suan Dusit University, Dusit, 10300 Bangkok, Thailand

**Keywords:** *Bacillus* sp., serine protease, halophilic bacteria, Vpr, structural modelling

## Abstract

**Research background:**

Haloalkaline proteases are one of the most interesting types of commercial enzymes in various industries due to their high specific activity and stability under extreme conditions. Biochemical characterization of enzymes is an important requirement for determining their potential for application in industrial fields. Most of microbial proteases have been isolated from *Bacillus* spp. In this study, the purification and characterization of an extracellular haloprotease produced from *Bacillus* sp. KB111 strain, which was previously isolated from mangrove forest sediments, are investigated for industrial applications.

**Experimental approach:**

The whole genome of KB111 strain was identified by DNA sequencing. Its produced protease was purified by salting out and anion-exchange chromatography, characterized based on protease activity and stability using a peptide substrate, and identified by LC-MS/MS.

**Results and conclusions:**

The strain KB111 was identified as *Bacillus licheniformis*. The molecular mass of its extracellular protease, termed KB-SP, was estimated to be 70 kDa. The optimal pH and temperature for the activity of this protease were 7 and 50 °C, respectively, while the enzyme exhibited maximal activity in the broad salinity range of 2–4 M NaCl. It was fully stable at an alkaline pH range of 7–11 at 50 °C with a half-life of 90 min. Metal ions such as K^+^, Ca^2+^ and Mg^2+^ could enhance the enzyme activity. Therefore, this protease indicates a high potential for the applications in the food and feed industry, as well as the waste management since it can hydrolyse protein at high alkaline pH and salt concentrations. The amino acid profiles of the purified KB-SP determined by LC-MS/MS analysis showed high score matching with the peptidase S8 of *B. licheniformis* LMG 17339, corresponding to the mature domain of a minor extracellular protease (Vpr). Amino acid sequence alignment and 3D structure modelling of KB-SP showed a conserved catalytic domain, a protease-associated (PA) domain and a C-terminal domain.

**Novelty and scientific contribution:**

A novel extracellular haloprotease from *B. licheniformis* was purified, characterized and identified. The purified protease was identified as being a minor extracellular protease (Vpr) and this is the first report on the halotolerance of Vpr. This protease has the ability to work in harsh conditions, with a broad alkaline pH and salinity range. Therefore, it can be useful in various applications in industrial fields.

## INTRODUCTION

Proteases (EC 3.4.21), also called peptidases, proteinases or proteolytic enzymes, are hydrolytic enzymes that hydrolyse peptide bonds within protein molecules. Proteases are classified into two major groups, exopeptidases and endopeptidases, based on the site of the cleaved peptide bond. Moreover, they are also classified as aspartic, glutamic, cysteine, metallo, serine, asparagine, threonine, mixed, unknown or compound peptidases, based on the catalytic type (*1*). The substrate specificity of proteases depends on the type of protease and the amino acid residues at the active site (*2*). For industrial enzymes, proteases constitute over 65% of the total enzyme market and have various applications, including food and feed industries, as laundry detergents and pharmaceuticals, and in waste management (*3*). Microbial proteases from extreme environmental conditions are scientifically and industrially significant due to their high specific activity and stability in broad ranges of pH, salinity and temperature (*4*). Currently, a lot of microbial proteases have been patented and commercialized, such as Alcalase, Savinase and Durazyme (Novozymes, Bagsværd, Denmark), Nagarase (Nagase, Osaka, Japan) and M-protease (Kao Corporation, Tokyo, Japan). Most of them are produced from *Bacillus* species (*5*, *6*).

Halophiles are microorganisms commonly found in saline environments and require salt for growth. They are a good source of useful salt-stable enzymes and important for industrial biotechnology due to their stability and activity in a broad salinity range. Halophilic microorganisms can be divided into three groups according to the salt concentration required for cell growth: slight (0.2–0.5 M NaCl), moderate (0.5–2.5 M NaCl) and extreme (2.5–5.2 M NaCl) halophiles (*7*). To prevent the diffusion of NaCl into cells in a high-salt environment, some anaerobic halophilic bacteria and aerobic halophilic archaea accumulate high concentrations of inorganic ions, especially K^+^, to balance osmotic pressure. In spite of this, eukaryotes and halophilic bacteria accumulate compatible solutes, the highly soluble organic compounds of small molecules, to maintain the concentration of NaCl in the cells (*8*). Halophilic proteases have a unique structure to catalyse hydrolysis reactions, such as an increase of acidic amino acid residues and a decrease of lysine residues or increase of small hydrophobic residues on the protein surface under hypersaline conditions (*9*). These proteases can be applied in the food industry in animal- and plant-based proteins such as fish, meat or soy under saline and saline-free conditions. Furthermore, they can be applied in biosynthetic processes, soil bioremediation and hypersaline waste treatment (*3*, *8*). Mostly, extracellular halophilic proteases have been isolated from *Bacillus* species and *Bacillus*-related genera such as *Bacillus luteus* H11 (*3*), *Bacillus* sp. EMB9 (*10*), *B. aquimaris* VITP4 (*11*) and *B. licheniformis* BA17 (*12*). For *Bacillus* strains, a lot of research has been done on alkaline proteases and thermophilic proteases, but very little research on halophilic and moderately halophilic proteases has been reported. Thus, information about their halotolerance is not easily accessible (*3*, *12*).

An extracellular protease produced by the moderately halophilic *Bacillus* sp. strain KB111 isolated from Chanthaburi, Thailand, under optimum conditions for the crude enzyme has been reported in a previous study (*13*). To apply this enzyme in biotechnological and industrial fields, in this study, the whole bacterial genome and the extracellular protease of *Bacillus* sp. KB111 strain were identified, the extracellular protease, termed KB-SP, was produced, purified and biochemically characterized, and the enzyme structure was predicted to understand the functional properties of the enzyme.

## MATERIALS AND METHODS

### Medium and chemicals

Nutrient broth and chemicals: Coomassie brilliant blue (CBB) R-250, 2-amino-2-(hydroxymethyl)propane-1,3-diol (Tris), Bradford reagent, bovine serum albumin (BSA) and acrylamide/bis-acrylamide solution were purchased from HiMedia (Mumbai, India). Low-molecular-mass marker was purchased from GeneDireX (Taoyuan, Taiwan). N-succinyl-Ala-Ala-Pro-Phe-*p*-nitroanilide (Suc-AAPF-*p*NA), azocasein, gelatin, 4-aminobenzamidine dihydrochloride, dithiothreitol, sodium dodecyl sulfate (SDS), glycerol, bromophenol blue, trichloroacetic acid (TCA), ethylenediaminetetraacetic acid (EDTA), Triton X-100, ammonium sulfate, sodium acetate, sodium phosphate, glycine, NaCl, NaOH, HCl, KCl, CaCl_2_, CuCl_2_, ZnCl_2_ and MgCl_2_ were purchased from Sigma-Aldrich, Merck (St. Louis, MO, USA).

### Microorganism and protease production

The protease-producing bacterium, termed KB111, used in this study was previously isolated (*13*) from mangrove forest sediments in Thailand. It exhibited a clear zone surrounding the colonies on skimmed milk agar and exhibited the highest protease activity in liquid medium. KB111 was inoculated in 5 mL of nutrient broth containing 0.5 M NaCl and cultured in a rotary shaker (WDS28; PolyScience, Niles, IL, USA) at 180 rpm and 37 °C. After that, cells were harvested for 8 to 64 h. The absorption of the cultures was checked at 600 nm by a UV spectrophotometer (UV-2401PC; Shimadzu, Kyoto, Japan). The cell cultures were harvested at 14 000×*g* (U-320R; Boeco, Hamburg, Germany) for 5 min at 4 °C and the crude enzyme supernatant was collected for the protease assay using azocasein as protein substrate. The reaction mixture containing 30 µL of the crude enzyme, 270 µL of 50 mM Tris-HCl (pH=7) and 20 mg/mL azocasein was incubated at room temperature (25 °C) for 30 min. Aliquots were withdrawn to determine the activity, as described in the enzymatic activity section.

### Whole bacterial genome sequencing

The genomic DNA of KB111 was extracted by Purelink^TM^ HQ mini plasmid purification (Invitrogen, Carlsbad, CA, USA) and the whole genome was sequenced by the Omics Sciences and Bioinformatics Centre, Bangkok, Thailand. The genomic DNA was processed according to Qiagen FX DNA library kit preparation (Hilden, Germany) and a library was sequenced by an Illumina MiSeq sequencer (San Diego, CA, USA) (paired-end 2×250 bp read length).

### Purification of protease

The crude protein supernatant of KB111 was collected for purification. The protease was precipitated by salting out using ammonium sulfate ranging from 40–50% (*m*/*V*) saturation. Then, the precipitate was recovered after centrifugation (U-320R; Boeco) at 14 000×*g* for 30 min, resuspended in 20 mM Tris-HCl (pH=8) and dialysed overnight against the same buffer. The protein solution was applied to a 5-mL diethylaminoethyl cellulose (DEAE) anion-exchange column (GE Healthcare, Marlborough, MA, USA) pre-equilibrated with 20 mM Tris-HCl (pH=8). The bound protein was subsequently eluted using a gradient of NaCl (0–1 M) in the same buffer. The protein fractions were collected, dialysed overnight against 20 mM Tris-HCl (pH=7) and then concentrated appropriately using a Centricon ultrafiltration unit (Millipore, Bedford, MA, USA) with a 10 kDa molecular cut-off. All purification stages were performed at 4 °C.

### SDS-PAGE and molecular mass determination

The protein sample was prepared by precipitation with 10% (by volume) TCA and dissolving the precipitate in SDS sample buffer, containing 50 mM Tris-HCl (pH=6.8), 2% (*m/V*) SDS, 0.1 M dithiothreitol, 10% (by volume) glycerol and 0.005% (*m/V*) bromophenol blue. After that, the solution was neutralized with 2 M NaOH and boiled for 5 min. The protein purity and molecular mass were determined by sodium dodecyl sulfate-polyacrylamide gel electrophoresis (SDS-PAGE) with 12% (*m/V*) polyacrylamide gel and the protein band was visualized by incubating the gel with staining solution (CBB R-250). The concentration of the purified protein was measured by the method of Bradford (*14*).

The protein molecular mass was determined by gel filtration column chromatography using a Sephacryl S-200HR column (GE Healthcare). Aldolase (158 kDa), conalbumin (75 kDa), ovalbumin (44 kDa) and ribonuclease (13.7 kDa) were used as protein markers.

### Activity staining of gel (zymography)

SDS-PAGE was performed using 12% (*m/V*) polyacrylamide gel containing 1 mg/mL gelatin. The sample was precipitated with 10% (by volume) TCA and boiled in SDS sample buffer for 5 min prior to loading onto the gel. Following electrophoresis, the gel was placed in 1% (by volume) Triton X-100 in phosphate-buffered saline for 2 h at room temperature (25 °C), then transferred into 50 mM Tris-HCl (pH=7) with 0.5 M NaCl for 10 h at room temperature (25 °C) and stained with CBB R-250. A clear zone at the location of the protease band was visualized because of the hydrolysis of gelatin.

### Enzymatic activity

Enzymatic activity was measured using a protein substrate (azocasein). The enzymatic reaction mixture containing 30 µL of an appropriate dilution of the enzyme, 270 µL of 50 mM Tris-HCl (pH=7), 0.5 M NaCl, and 20 mg/mL azocasein was incubated for 20 min. After that, 200 µL of 15% (by volume) TCA were added to stop the enzymatic reaction. The mixture was kept on ice for 10 min and centrifuged (U-320R; Boeco) at 14 000×*g* for 15 min. The supernatant (160 µL) was neutralized by mixing with 40 µL of 2 M NaOH. The absorbance of the resulting solution was measured (UV-2401PC; Shimadzu) at 440 nm. The enzymatic activity of the sample was determined in units (U), which was the amount of enzyme in the assay reaction mixture that increased the absorbance at 440 nm by 0.1 in 1 min.

Specific enzymatic activity was measured using a peptide substrate (Suc-AAPF-*p*NA). The enzymatic reaction mixture containing 2 µL of an appropriate dilution of the enzyme, 98 µL of 50 mM Tris-HCl (pH=7), 0.5 M NaCl and 2 mM Suc-AAPF-*p*NA was incubated for 20 min. The absorbance of *p*-nitroaniline released by enzymatic hydrolysis was measured at 410 nm with a molar absorption coefficient of 8900 M^−1^ cm^−1^. The enzymatic activity of the sample was determined in U, which was the amount of enzyme in the assay reaction mixture that produced 1 µmol of *p*-nitroaniline in 1 min. The specific activity of the enzyme was defined in U of the enzyme per milligram of protein.

### Effects of pH, temperature, NaCl and metal ions on protease activity

The optimum pH was examined at various pH ranging from 4.5 to 10 at 30 °C. Different buffers (50 mM) with 0.5 M NaCl were used in the assay reaction mixtures: sodium acetate (pH=4.5–5.5), sodium phosphate (pH=5.5–7), Tris-HCl (pH=7–9) and glycine-NaOH (pH=9–10). The optimum temperature was examined by incubating the enzyme in 50 mM Tris-HCl (pH=7) with 0.5 M NaCl at various temperatures ranging from 20 to 80 °C. For the effect of NaCl on protease activity, the enzyme was dissolved in 50 mM Tris-HCl (pH=7) and different concentrations of NaCl ranging from 0 to 4 M. To determine the effect of metal ions, KB-SP was pre-incubated for 3 h at 30 °C with 50 mM Tris-HCl (pH=7) with different metal ions: KCl, CaCl_2_, CuCl_2_, ZnCl_2_ and MgCl_2_ (final concentration of 10 mM). Enzymatic activity was measured using Suc-AAPF-*p*NA and expressed in percentage of relative activity by taking the highest activity obtained at optimal pH, temperature and NaCl as 100% and by taking the control (without metal ions) as 100% for metal ions.

### Determination of kinetic constants

The kinetic parameters of the enzyme were assayed using Suc-AAPF-*p*NA of 0.5–4 mM as a substrate in 50 mM Tris-HCl (pH=7) with 2 M NaCl. The reaction followed the Michaelis–Menten kinetics, the *v*_max_ and *K*_m_ values were obtained using Lineweaver–Burk plots. The *k*_cat_ value was obtained by dividing the *v*_max_ value with the concentration of enzyme.

### Effects of pH, temperature and EDTA on KB-SP stability

The pH stability of KB-SP was determined by pre-incubating the enzyme for 24 h at various pH ranging from 1 to 12.5 at 30 °C. Different buffers (50 mM) with 0.5 M NaCl were used in the reaction mixture for pre-incubation: KCl-HCl (pH=1–1.5), glycine-HCl (pH=2-3), sodium acetate (pH=4–5), sodium phosphate (pH=6), Tris-HCl (pH=7–9), and glycine-NaOH (pH=10–12.5). The residual activity of the enzyme was analysed at 30 °C using Suc-AAPF-*p*NA in 50 mM Tris-HCl (pH=7), with 2 M NaCl. To analyse the thermal stability of KB-SP, the enzyme was incubated in 50 mM Tris-HCl (pH=7), with 0.5 M NaCl at 40, 50 and 60 °C. The residual activity of KB-SP was analysed at 30 °C using Suc-AAPF-*p*NA in 50 mM Tris-HCl (pH=7) with 2 M NaCl. To determine the effect of EDTA treatment on its stability, KB-SP was incubated at 40 and 50 °C in 50 mM Tris-HCl (pH=7) with 10 mM EDTA and the residual activity was analysed at 30 °C using Suc-AAPF-*p*NA.

### Protein identification by LC-MS/MS

The purified protein, termed KB-SP, was excised as a single band from the SDS-PAGE gel and dehydrated. After drying, the presence of the amino acids on the gel was analysed using liquid chromatography–tandem mass spectrometry (LC-MS/MS) by First BASE Laboratories, Selangor, Malaysia. The sample was digested with trypsin and extracted into peptides following standard methods (*15*). Peptides were analysed using a nano HPLC-MS/MS system (Shimadzu), containing a triple time-of-flight (TOF) mass spectrometer model TripleTOF® 5600+ (Sciex, Framingham, MA, USA) *via* electrospray ionization (ESI) interface. The tryptically digested peptides were injected into an Agilent Zorbax 300SB-C18 column, 3.5 µm particle size (Agilent Technologies, Santa Clara, CA, USA) to separate peptides by linearly increasing the gradient of *φ*(formic acid, water)=0.1%/acetonitrile. Protein spectra were analysed and identified using Mascot sequence matching software (*16*) and the UniProt database (*17*) with the taxonomy of the genus *Bacillus*.

### Prediction of structure

The amino acid sequence of KB-SP was used to generate the homology or comparative 3D modelling structure using the Expasy SWISS-MODEL server (*18*). For model building, the template was selected based on the highest quality of the QMEAN score. After modelling, the structural validation of the model was checked and analysed by PROCHECK using the PDBsum server (*19*), and the secondary structure of the protein was predicted using the ProFunc server (*20*). The 3D images of structures to analyse the location of the amino acid residues were generated using PyMOL software (v. 2.4) (*21*).

### Statistical analysis

All experiments in this study were carried out in three replicates and the data represent the mean value±standard error. The SPSS software v. 22 (*22*) was used for statistical analysis by one-way ANOVA and differences in the mean values were subjected to Duncan’s multiple range test at 0.05 level (p≤0.05).

## RESULTS AND DISCUSSION

### Production of protease

Strain KB111 was isolated from mangrove forest sediments in Chanthaburi, Thailand. The isolate was examined and further identified as a Gram-positive, rod-shaped bacterium, and its protease production was optimized on skimmed milk agar containing 30 mg/mL NaCl by a point inoculation technique and in liquid medium using azocasein as substrate. Based on 16S rRNA gene sequence analysis, the isolate KB111 was determined as being closely related to *Bacillus* sp. (*13*). Furthermore, the whole genomic DNA of KB111 was identified, and it displayed average nucleotide identity (ANI) of 99.68% with *Bacillus licheniformis* LMG 17339. Strain LMG 17339 was isolated from silage in Denmark by the Danish Veterinary Laboratory and was grown aerobically on nutrient agar or trypticase soy agar at 37 °C (*23*). Its genome contains 21 genes encoding proteases/peptidases (*24*). However, no research on purification and characterization of the proteases/peptidases in this genome has been reported. The *B. licheniformis* protease termed subtilisin Carlsberg was the first bacterial protease used in detergent, in the 1960s. More recently, a large group of commercial alkaline proteases have been derived from *Bacillus* strains, such as *B. amyloliquefaciens*, *B. licheniformis* and *B. clausii*. For example, Alcalase and Savinase from Novozymes are industrial alkaline proteases from *B. licheniformis* and *B. clausii*, respectively (*25*). Furthermore, carbohydrase and protease enzymes of *B. licheniformis* strains are listed in the fourth edition of the Food Chemicals Codex for application in food processing (*26*).

**Fig. 1:**
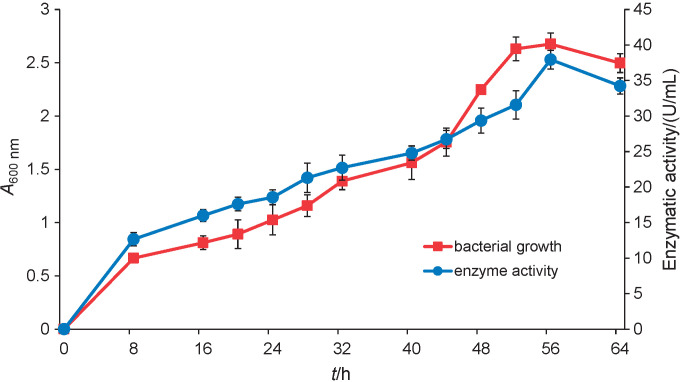
Time course of growth and protease production of *Bacillus licheniformis* KB111. Enzymatic activity was determined from crude enzyme supernatant using azocasein as a substrate

The relationship of KB111 protease activity with bacterium growth was examined during incubation at 37 °C for 8 to 64 h using azocasein as a substrate. Azocasein is a non-specific protease substrate and is normally used to determine the proteolytic activity of proteases. The result shows that KB111 exhibited the highest protease activity ((38.0±1.3) U/mL) after 56 h of incubation, at the end of the stationary phase, and it gradually decreased during the stationary phase (Fig. 1). This result corresponds to those for the haloalkaline protease from *Bacillus* sp. SM2014 (*27*) and the alkaliphilic strain *B. pumilus* MCAS8 (*28*). In addition, most *Bacillus* spp. exhibit their highest protease activity during the post-exponential and stationary phases (*29*, *30*). These results indicate the role of the extracellular protease of this organism in survival during the stationary phase in the environment ecology (*29*).

### Protease purification

**Fig. 2:**
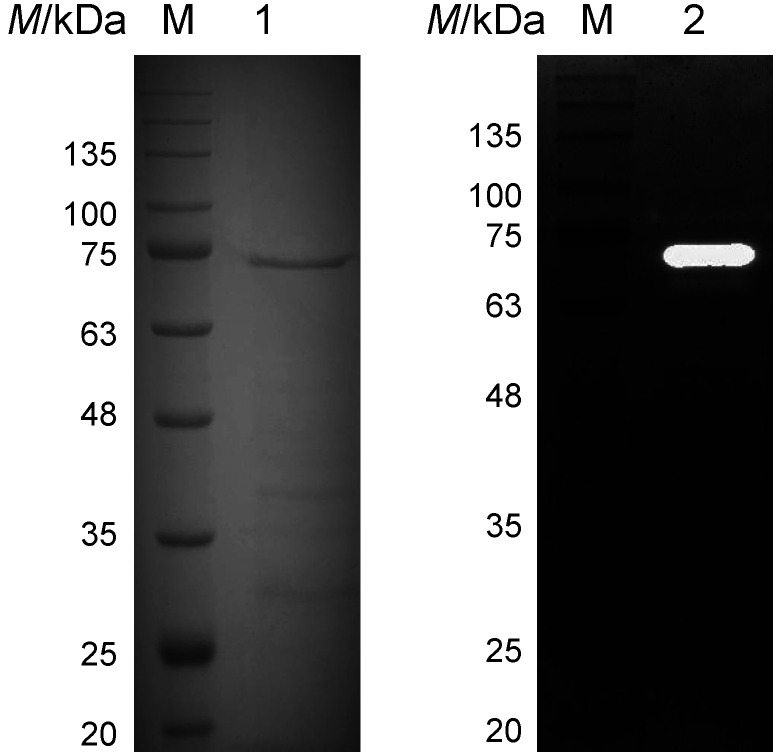
Analysis of purified KB-SP by SDS-PAGE (lane 1) and activity staining of gel (lane 2). Lane M=low-molecular-mass marker, lanes 1 and 2=purified KB-SP protein

The extracellular protease KB-SP was purified by the addition of ammonium sulfate and HiTrap DEAE (anion-exchange chromatography). The enzymatic activity in each step was determined using 2 mM Suc-AAPF-*p*NA at 30 °C in 50 mM Tris-HCl (pH=7). Suc-AAPF-*p*NA as a synthetic peptide peptidyl-*p*NA was used to determine the enzymatic activity because the extracellular serine protease and alkaline protease showed the highest specificity towards Suc-AAPF-*p*NA (*31*, *32*). KB-SP was purified 8.6-fold with a (20.1±0.2) % yield and a specific activity of (15.0±0.2) U/mg in the final purification step (Table 1). The total mass of KB-SP was (2.8±0.1) mg purified from 1 L of culture. In SDS-PAGE analysis, the purified KB-SP showed a major activity band at 70 kDa by both CBB staining and zymography and some weak bands at 38 and 28 kDa in CBB staining (Fig. 2). KB-SP was denatured by TCA treatment and boiling in the presence of SDS prior to SDS-PAGE. However, KB-SP exhibited activity in the gel (zymography) probably because it is partially refolded after dialysis against 1% (by volume) Triton X-100 in phosphate-buffered saline. Protease activity in the gel was also observed in a previous study (*33*). By gel filtration column chromatography, the molecular mass of purified KB-SP was calculated to be 68 kDa, which corresponds to a major band on SDS-PAGE, suggesting that KB-SP is a monomer.

**Table 1:** Purification of KB-SP from *B. licheniformis* strain KB111 (1-litre fermentation medium)

Purification step	Total activity/ U	*m*(total protein)/mg	Specific activity/ (U/mg)	Purification fold	*Y*/%
Crude supernatant	209±14	120±12	1.7±0.1	1.0±0.0	100.0±0.0
Ammonium sulfate precipitation	76.6±2.3	14.4±1.2	5.3±0.2	3.06±0.09	36.7±1.1
DEAE column chromatography	41.9±0.4	2.8±0.1	15.0±0.2	8.60±0.09	20.1±0.2

DEAE=diethylaminoethyl cellulose

### Protease activity

**Fig. 3:**
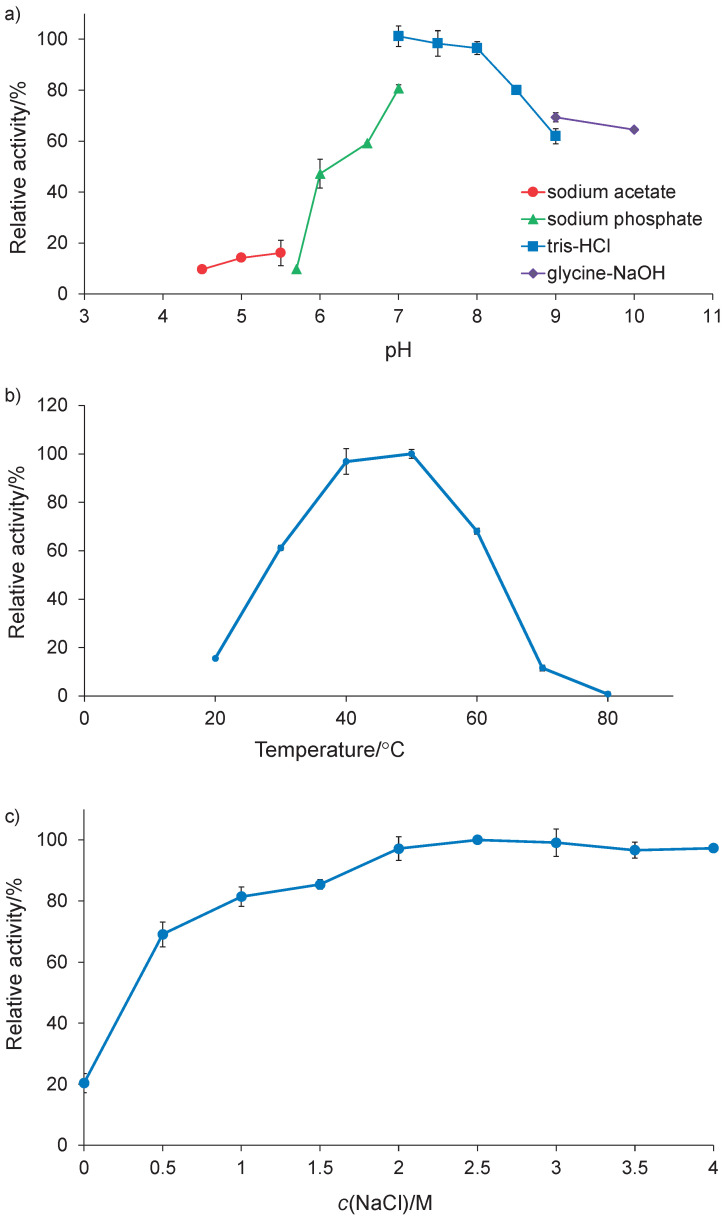
Effects of: a) pH, b) temperature, and c) NaCl on protease activity. Enzymatic activity was assayed using Suc-AAPF-*p*NA as a substrate

The purified KB-SP exhibited more than 50% of the highest activity at pH=6.5–10, the optimum being at pH=7 in 50 mM Tris-HCl. However, at pH=7 and 8 the activity was at 95% (Fig. 3a). Considering temperature dependence, KB-SP exhibited the highest activity at 50 °C, but it did not differ significantly from that at 40 °C. The protease exhibited approx. 60 and 70% of the highest activity at 30 and 60 °C, respectively (Fig. 3b). This result corresponds with a previous study in which the crude enzyme of isolate KB111 was determined, with its optimal pH=7.0 and temperature of 40 °C with azocasein as substrate (*13*). This result is similar to that for Neutrase 0.8L (Novozymes) with optimal activity at pH=7 and 40–50 °C (*34*).

Considering the effect of NaCl on protease activity, KB-SP exhibited high activity in the broad salinity range of 2–4 M NaCl and lost 80% of its activity in the absence of NaCl (Fig. 3c). This result suggests that KB-SP is a halotolerant protease. It has been reported in a previous study that isolate KB111 can grow at NaCl concentrations ranging from 0.5 to 1.5 M. It exhibits activity in the presence of 0–2 M NaCl (*13*), a range similar to those observed for the halotolerant *B. licheniformis* BA17 (0–3.4 M NaCl) (*12*) and *B. licheniformis* TD4 (0–2 M NaCl) (*35*). However, *B. licheniformis* BA17 and TD4 have not yet been studied to determine the effect of NaCl on the protease activity of the purified enzyme. The high activity of purified KB-SP in a broad salinity range was similar to that of the alkaline and halophilic proteases produced by *B. luteus* H11 at 1–5 M NaCl (*3*). Nevertheless, the effect of NaCl concentration on KB-SP activity was superior to that on the activity of other *Bacillus* spp. haloalkaline proteases because approx. 80% of the activity of KB-SP was retained at 1–4 M NaCl, while more than 80% of the activity of *Bacillus* sp. SM2014, *Bacillus* sp. APCMST-RS7 and *B. iranensis* X5B was retained at 0–2, 1–1.5 and 0.5–1.5 M NaCl, respectively (*27*, *36*, *37*). In a previous study, halophilic proteases were obtained from *Bacillus* spp., *Halobacillus* spp., *Salinivibrio* sp., *Pseudoalteromonas* sp., *Filobacillus* sp., *Virgibacillus* sp. and *Chromohalobacter* sp. (*9*). Halophiles can live in an environment with high salt concentration due to their adaptation mechanism which prevents the diffusion of NaCl into the cells and unique structural features of proteins to maintain their function under high-salt conditions (*8*, *10*).

Considering the effect of metal ions, the enzymatic activity of KB-SP in the presence of the divalent cations Ca^2+^ and Mg^2+^ and monovalent cation K^+^ at a final concentration of 10 mM increased by 22, 25 and 30%, respectively, when compared to the control (Table 2). The activity of control was determined after dialysis against 10 mM EDTA. These results are similar to those reported for *B. licheniformis* MP1 and BA17 for each metal ion at 5.0 mM (*32*, *38*). In addition, the enzymatic activity of *B. licheniformis* RP1 and P003 and halophilic proteases from *Bacillus* spp. such as *B. luteus* H11 and *B. iranensis* X5B had also been found to increase with the addition of Ca^2+^ and Mg^2+^ ions at a final concentration of 1.0–5.0 mM (*3*, *30*, *37*, *39*). Nevertheless, ZnCl_2_ and CuCl_2_ had a negative effect on KB-SP activity. It was inhibited by 80 and 97% in the presence of Cu^2+^ and Zn^2+^ ions, respectively. These results are similar to those reported for *B. luteus* H11, *B. licheniformis* RP1, and *Bacillus* sp. APCMST-RS7 for each metal ion at 1.0 and 5.0 mM (*3*, *30*, *36*). The results of metal ion influence indicated that KB-SP could be used in shrimp waste powder because it contained 13.45, 0.58, 0.12 and 0.07% of Ca^2+^, Na^+^, Mg^2+^ and K^+^ ion, respectively (*30*), which markedly affected the increased KB-SP activity.

**Table 2:** Effect of metal ions on the activity of KB-SP

Metal ion	Relative activity/%
Control KCl CaCl_2_ CuCl_2_ ZnCl_2_ MgCl_2_	(100)^b^ (129.8±2.7)^a^ (121.5±5.5)^a^ (19.7±0.6)^c^ (2.9±1.6)^d^ (124.5±2.7)^a^

*c*(metal ion)=10 mM. Mean values±standard error with different superscript are significantly different (p≤0.05)

The kinetic parameters of KB-SP were determined at 30 and 50 °C using Suc-AAPF-*p*NA as a substrate. The *v*_max_ value of KB-SP at 30 and 50 °C was (23.5±1.1) and (47.7±2.9) U/mg, respectively (data not shown). The *K*_m_ value of KB-SP was rather similar at 30 °C (1.0±0.2 mM) and 50 °C (1.0±0.1 mM), indicating the same substrate binding affinity at both temperatures. However, the *k*_cat_/*K*_m_ values of KB-SP at 30 °C ((25.6±2.4) s^−1^ mM^−1^) were considerably lower than at 50 °C ((54.1±2.3) s^−1^ mM^−1^) (data not shown). The results show that KB-SP was characterized by a low turnover number and catalytic efficiency at low temperatures.

### Stability

**Fig. 4:**
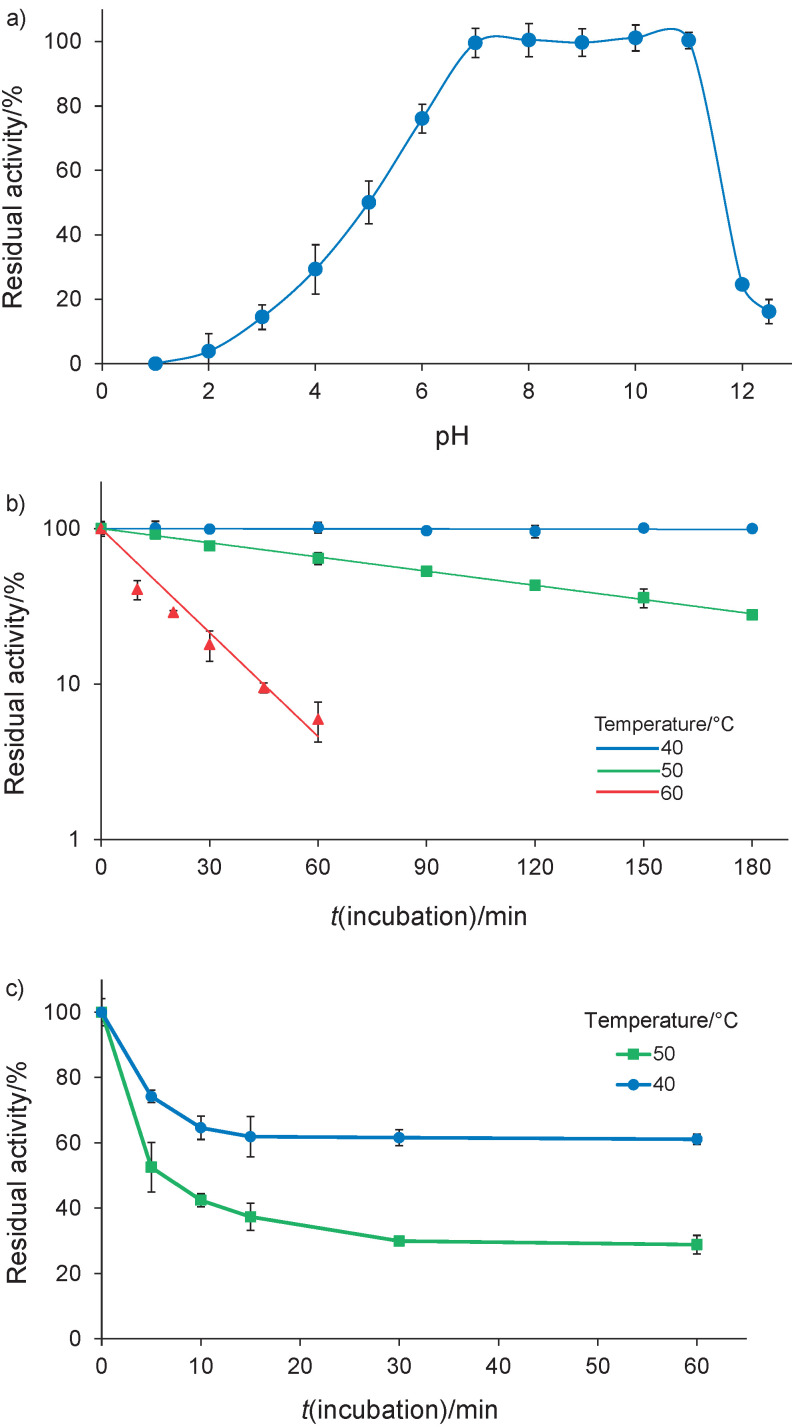
Effects of: a) pH, b) temperature, and c) EDTA on protease stability. Residual activity was determined at 30 °C using Suc-AAPF-*p*NA as a substrate. For thermal stability against irreversible heat inactivation, residual activity was plotted on a semilog scale against incubation time (b)

The purified KB-SP was fully stable at pH=7–11, unlike at pH≤6 and ≥12. It lost >75% of its activity at pH≤3 and ≥12 (Fig. 4a). The effect of pH on the stability and activity of KB-SP was similar to the findings of previous studies on proteases from other bacteria having a pH=7–12 optimal for haloalkaline proteases (*3*, *27*, *32*, *40*) and the alkaline protease belonging to *B. licheniformis* (*38*, *39*, *41*, *42*). However, KB-SP was more stable at alkaline pH than the commercial enzymes subtilisin Carlsberg and Alcalase from *B. licheniformis*, which show pH stability at 8–10 and 6–10 (*34*), respectively.

The results for KB-SP against irreversible heat inactivation showed that it was stable for at least 3 h at 40 °C and it lost activity with a half-life of 90 and 10 min at 50 and 60 °C, respectively (Fig. 4b). This result confirms that KB-SP is active and stable in the mesophilic temperature range. The optimum temperature and thermal stability of KB-SP were found to be similar to those of the halotolerant *B. licheniformis* BA17 with a half-life of 90 and 12 min at 50 and 60 °C, respectively (*32*). In general, alkaline proteases from *B. licheniformis* and halophilic proteases from other bacteria remain active and stable at temperatures ranging between 40 and 70 °C (*38*, *41*, *43*, *44*).

Considering the effects of EDTA treatment on stability, KB-SP lost approx. 40% of activity by incubation at 40 °C for 10 min and rapidly lost activity with a half-life of 5 min at 50 °C. These results suggest that the stability of KB-SP can be lost in the presence of EDTA (Fig. 4c). EDTA is a chelating agent that can bind to metal ions to form a complex in the molecule. Therefore, metal ions are required for the maximum stability of KB-SP; these ions tightly bind to the enzyme and are difficult to remove in the absence of EDTA.

The results of biochemical characterization indicate that KB-SP may be useful in various applications in industry at relatively high alkaline pH and salt concentrations. KB-SP can be applied in the food industry as a milk-clotting enzyme to produce milk curd (*45*) and protein hydrolysate using fish, meat and soy as raw material (*46*). Furthermore, it can be used in the feed industry as an additive that increase the protein digestibility of high-protein meal such as soybean (*47*), and for deproteinization of waste in the seafood industry (*38*).

### Identification of KB-SP

**Fig. 5:**
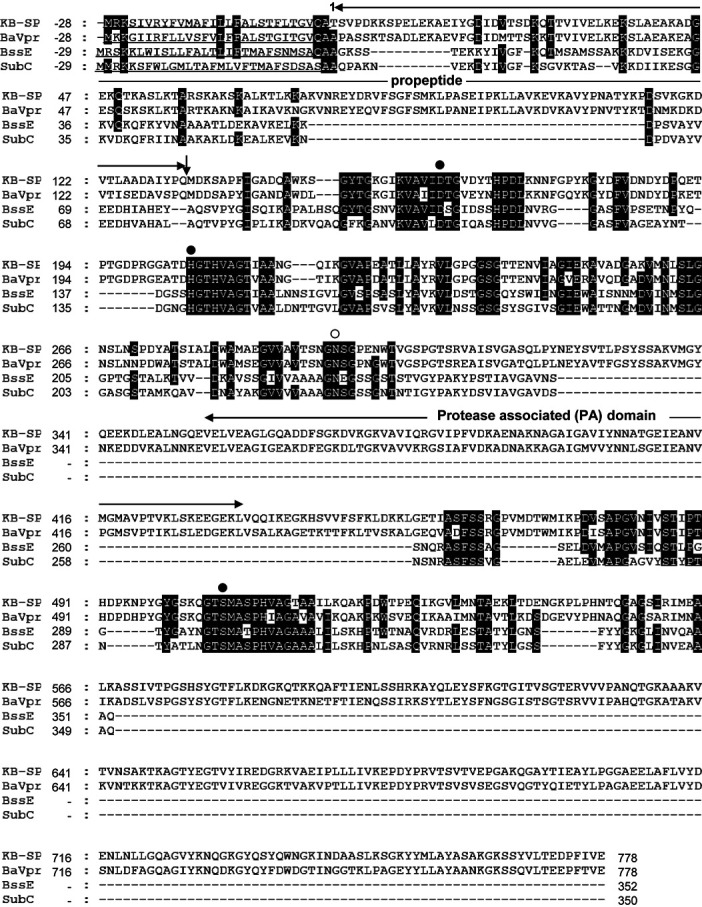
Amino acid sequence alignment of serine proteases. The amino acid sequence of Prepro-KB-SP (KB-SP) is compared with those of Prepro-*Bacillus* Vpr (BaVpr), Prepro-subtilisin E (BssE) and Prepro-subtilisin Carlsberg (SubC). Dashes represent gaps in the sequence. Black highlights indicate homologous amino acid residues in the three least different proteins. Solid circles indicate a catalytic triad consisting of Asp, His and Ser residues and open circles indicate an oxyanion hole. Signal sequences (underlined) were experimentally determined for BssE (*48*) and SubC (*49*) or estimated for KB-SP and BaVpr using the SignalP 5.0 Server (*50*). Filled arrows represent the position of the propeptides of Pro-BaVpr, Pro-BssE and Pro-SubC, which are autoprocessed. Accession numbers: KUL11341 for KB-SP, AAA22881 for BaVpr, AAA22742 for BssE and X03341 for SubC

To determine what type of enzyme it is, the purified KB-SP was incubated with the serine protease inhibitor, 4-aminobenzamidine dihydrochloride (final concentration of 10 mM) and its protease activity was determined by zymography. No activity band was detected by zymography, indicating that KB-SP belongs to the serine protease family. In addition, KB-SP retained more than 60% of its activity when incubated with 10 mM EDTA, a metalloprotease inhibitor (Fig. 4c), indicating that KB-SP is not a metalloprotease. After that, the amino acid sequence of purified KB-SP was identified by LC-MS/MS as RVVVPANQTGKA, RVTSVTVEPGAKQ, KGVAPEATLLAYRV, RGVIPFVDKAENAKN, *i.e.* with a high score matching with that of peptidase S8 (accession no. KUL11341) of *B. licheniformis* LMG 17339. This 2421 bp gene encodes 806 amino acid residues with a molecular mass of 86 kDa. The amino acid sequence of this gene contains a signal sequence, a propeptide and a mature domain, termed Prepro-KB-SP, and it was compared with those of the serine protease Vpr from *B. subtilis* (accession no. AAA22881), termed Prepro-BaVpr, in this study (Fig. 5 (*48*-*50*)). Based on amino acid sequence identities, Prepro-KB-SP displayed 72.21% similarity to Prepro-BaVpr. The signal sequences for protein secretion from the cell membrane were estimated for Prepro-KB-SP and Prepro-BaVpr using SignalP 5.0 (*50*) or experimentally determined for BssE (*48*) and SubC (*49*) and are underlined in Fig. 5. In a previous study, the propeptide BaVpr was determined as being composed of 132 amino acids and processed from a propeptide form upon cleavage of the peptide bonds between Gln132 and Met133 (*51*). These amino acids are also conserved in KB-SP, probably because KB-SP comprises a propeptide form (Pro-KB-SP, Thr1-Gln132) and a mature domain (Met133-Glu778). The mature domain was calculated from the amino acid sequence as having a mass of 68 kDa, which corresponds to the activity staining of the gel (zymography) of KB-SP with a molecular mass of ~70 kDa. Therefore, the purified enzyme may represent the mature domain of KB-SP. Furthermore, weak bands of 38 and 28 kDa were observed on SDS-PAGE (Fig. 2), corresponding to the previous result for BaVpr (*51*). These weak bands may represent the proteolytic fragment of the mature domain. The three residues of a serine protease that form a catalytic triad are completely conserved in the KB-SP sequence as Asp161, His205 and Ser516, and the asparagine residue that forms an oxyanion hole is also conserved as Asn295. Between the histidine and serine residues of the active site, KB-SP and BaVpr have an unusual 132 amino acid residue, longer than the other serine protease of *B. subtilis* (subtilisin E; BssE) and *B. licheniformis* (subtilisin Carlsberg; SubC) (Fig. 5). Vpr was identified as a minor extracellular protease with an Asp/His/Ser catalytic triad, which is similar to that found in the active site of subtilisin (*51*, *52*). Collagen, fibrin, casein, gelatin and albumin can be used as protein substrates for Vpr (*53*). Vpr from *B. subtilis* and *B. licheniformis* has been identified as a fibrinolytic enzyme and a milk-clotting protease, respectively (*45*, *54*). Vpr contains an N-terminal signal sequence, a propeptide, a catalytic domain (peptidase S8) and a C-terminal protease-associated (PA) domain (*52*). The PA domain is also conserved in KB-SP with 73.75% amino acid sequence similarity to BaVpr. According to a previous study, the long C-terminal PA domain has a high molecular substrate-binding specificity (*53*). The Vpr from *Bacillus* sp. has been found to have an optimal pH=7–8.5 and temperature of 37–50 °C (*45*, *52*, *53*), close to those of KB-SP at pH=7 and 50 °C. However, no information is available about the effect of NaCl on its activity. Therefore, this is the first report indicating that Vpr exhibits maximum activity in the presence of NaCl. However, further functional and structural studies are necessary to understand the autoprocessing, folding and substrate binding of KB-SP.

### 3D modelling

**Fig. 6:**
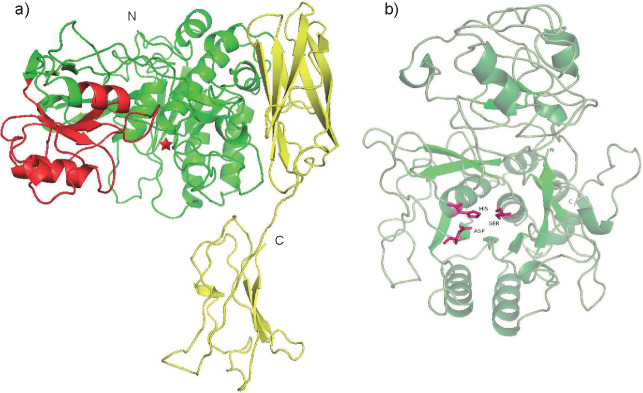
3D model of KB-SP created using Expasy SWISS-MODEL (*18*) in cartoon representation: a) the main-chain folding of KB-SP and b) the catalytic domain. The catalytic domain (Met133-Glu353 and Val434-Ser569), protease-associated domain (Val354-Leu433) and C-terminal domain (Ser570-Glu778) are coloured green, red and yellow, respectively. The position of the active sites is indicated with a red star and the active sites Asp, His and Ser are shown as purple stick models. N and C=N- and C-terminus, respectively

Due to the lack of information about the structure of Vpr in the Protein Data Bank (PDB) (*19*), the amino acid sequence of the mature domain (KB-SP) was used to generate a homology model using the SWISS-MODEL server (*18*). KB-SP displayed maximum sequence similarity of 26.68% to C5a peptidase (ScpA, a multidomain cell-envelope subtilase) from *Streptococcus pyogenes* [3eif.1.A]. Thus, ScpA was used as a template to predict the 3D structure of KB-SP. The model of the KB-SP structure was visualized and analysed using PyMOL software (*21*), as shown in Fig. 6a. The KB-SP structure consists of 13 β-sheets and 12 α-helices in the catalytic domain, 4 β-sheets and 3 α-helices in the PA domain, and 10 β-sheets and 1 α-helix in the C-terminal domain. Similar to all members in the serine protease family, KB-SP contains a catalytic triad consisting of three active site residues, Asp161, His205 and Ser516 (Fig. 6b). Folding of the main chain of the catalytic domain of KB-SP is related to that of the subtilisin domain with the subtilisin-like α/β domain, but the PA domain is not available in bacterial subtilisins. However, ScpA contains a PA domain with 8 β-sheets and 2 α-helices and has 37.66% amino acid sequence similarity to the PA domain of KB-SP.

## CONCLUSIONS

The *Bacillus licheniformis* strain KB111 isolated from mangrove forest sediments in Chanthaburi, Thailand, has the capability to produce a serine protease of ~70 kDa (KB-SP). This enzyme has the ability to work in harsh conditions, with a broad alkaline pH and salinity range. Its activity increases in the presence of metal ions K^+^, Ca^2+^ and Mg^2+^. These properties of the enzyme indicate that it is worth exploring its applicability in the food industry and for shrimp waste deproteinization. The purified KB-SP was identified as an extracellular protease, Vpr, containing a catalytic domain, a protease-associated domain and a C-terminal domain. This report appears to be the first on the minor extracellular halotolerant protease Vpr from *B. licheniformis*. However, further studies on the effect of organic solvents and structural insights are necessary to apply this enzyme in biotechnology and industry fields.
